# Cerebral Blood Flow Is Not a Direct Surrogate of Behavior: Performance Models Suggest a Role for Functional Meta-Networks

**DOI:** 10.3389/fnins.2022.771594

**Published:** 2022-02-15

**Authors:** John J. Sidtis

**Affiliations:** ^1^Brain and Behavior Laboratory, The Nathan Kline Institute for Psychiatric Research, Orangeburg, NY, United States; ^2^Department of Psychiatry, New York University Langone School of Medicine, New York, NY, United States

**Keywords:** cerebral blood flow, positron emission tomography, performance-based analysis, speech, spino-cerebellar ataxia, Parkinson’s disease, cortical-subcortical circuits

## Abstract

**Background:**

Functional brain imaging has become the dominant approach to the study of brain-behavior relationships. Unfortunately, the behavior half of the equation has been relegated to second-class status when it is not ignored completely. Different approaches to connectivity, based on temporally correlated physiological events across the brain, have ascended in place of behavior. A performance-based analysis has been developed as a simple, basic approach to incorporating specific performance measures obtained during imaging into the analysis of the imaging data identifying clinically relevant regions.

**Methods:**

This paper contrasts performance-based lateralized regional cerebral blood flow (CBF) predictors of speech rate during Positron Emission Tomography with the values of these regions and their opposite hemisphere homologs in which a performance-based model was not applied. Five studies were examined: two that utilized normal speakers, one that utilized ataxic speakers, and two that examined Parkinsonian speakers.

**Results:**

In each study, the predictors were lateralized but the blood flow values that contributed to the performance-based analysis were bilateral. The speech-rate predictor regions were consistent with clinical studies on the effects of focal brain damage.

**Conclusions:**

This approach has identified a basic, reproducible blood flow network that has predicted speech rate in multiple normal and neurologic groups. While the predictors are lateralized consistent with lesion data, the blood flow values of these regions are neither lateralized nor distinguished from their opposite hemisphere homologs in their magnitudes. The consistent differences between regional blood flow values and their corresponding regression coefficients in predicting performance suggests the presence of functional meta-networks that orchestrate the contributions of specific brain regions in support of mental and behavioral functions.

## Introduction

Behavioral physiologists at the end of the 19th century viewed the central nervous system in terms of strict stimulus-response interactions. While this appeared to be appropriate for basic sensory and motor functions, the dawn of the 20th century revealed problems for this approach in characterizing complex behaviors. One of the problematic situations arose from the judgments of various qualities of stimuli. The stimulus-response explanation predicted that while errors could occur, the perceived quality would be roughly veridical with the actual quality of the stimulus. However, it was reliably observed that the judgment of a stimulus’ quality, its weight for example, was influenced not only by the quality of a previous judged stimulus but by the qualities of all of the items in the stimulus set. This and other similar observations supported the notion of mental set, or “Einstellung,” a concept that introduced a psychological or cognitive element to the judgment process (Woodworth, 1938). While this psychological explanation became influential in the accounts of many types of behavior through the first half of the 20th century (Gibson, 1941), the mechanism was psychological rather than physiological.

Modern functional imaging has become quite sophisticated in measuring physiological signals like cerebral blood flow (CBF) as a measure of brain, neuronal, and as is often assumed, mental activity. The ability to measure CBF and cerebral metabolism, combined with the desire to explore the brain’s functions, ironically led to a growing stimulus-response conceptualization analogous to that employed by the 19th century physiologists. Simply put, if CBF increased in a region, that area of the brain was assumed to be involved in the mental or behavioral task in which the subject was involved. Much of the field has moved to assessing the “functional connectivity” across multiple brain areas, an approach that largely replaces relatively simple CBF increases with a more complex temporal correlation pattern of CBF across multiple regions (Friston, 2011). Subjects need not be engaged in any task for functional connectivity to be assessed. It is generally assumed that such temporal patterns can represent broad networks that identify a general condition of the brain like the resting state (Damoiseaux and Greicius, 2013), developmental issues (Cerliani et al., 2015), or pathological conditions (Allen et al., 2007; Baker et al., 2014). Functional connectivity has the capacity to distinguish pathological conditions on a global neurophysiological scale but it has often become the response in the stimulus-response paradigm with the strength of connections replacing the magnitude of CBF or cerebral metabolism as surrogate markers of mental function.

A different approach has been developed to better understand the relationship between brain activity and specific behaviors: performance-based analysis (Sidtis et al., 2003; Sidtis, 2007, 2012). This approach does not rely on contrasting CBF patterns for different tasks to attempt to identify specific brain-behavior relationships. In the case of speech production, neither adding nor subtracting CBF images of spoken syllables, phonated vowels, or articulatory gestures produce maps that in any way represent the known mapping of speech (Sidtis et al., 1999). The performance-based analysis does not rely on temporal correlation either. Instead, a specific task performance measure is obtained during scanning and multiple linear regression is used to assess whether a pattern of CBF is predictive of the performance measured. This approach has identified a modest, reproducible cortical-subcortical network associated with speech rate, one characteristic of a complex behavior. It has also identified complementary networks for different language modes (Sidtis et al., 2018; Van Lancker Sidtis and Sidtis, 2018a,b). A major advantage of performance-based analysis is that it identifies networks that include regions consistent with lesion data (Sidtis, 2012; Van Lancker Sidtis and Sidtis, 2018a,b).

In the first application of a performance measure to image data obtained from normal speakers (Sidtis et al., 2003), several analytic approaches to relating regional CBF to speech rate were examined. There were no significant relationships between speech rate and the following measures: un-normalized CBF, speech–rest CBF differences, and a scaled subprofile model (SSM) covariance analysis (Moeller et al., 1987) of speech–rest CBF differences.

However, two analytic approaches did result in linear regression models that were significantly associated with speech rate. An SSM analysis of speech scan CBF alone (no rest state contrast) produced a predictive model consisting of the subject scaling factors of seven regions: the left and right caudate, the left and right inferior frontal regions, the left putamen, and left and right cerebellar regions. The scaling factors were negatively weighted for the left and right caudate and inferior frontal regions, and both cerebellar regions. The left putamen was the only positively weighted. A significant linear regression relationship with speech rate was also observed with the normalized CBF identifying two regions: a positive contribution by the left inferior frontal region and a negative contribution by the head of the right caudate nucleus. The magnitudes of neither the unnormalized or normalized CBF measures of the predictor regions were significantly greater than the responses in their homologous regions in the opposite hemisphere, nor were the predictor regions among those with the greatest blood flow responses.

It was clear from these analyses that subtracting resting state values did not facilitate a solution to predicting speech rate. While both the SSM subject factor analysis and the normalized CBF analysis produced models that were significantly correlated with speech rate, the normalized CBF linear regression approach was pursued for two reasons. First, the lateralized inferior frontal and caudate region solution was consistent with the results of lesion studies. Second, a more direct relationship between CBF and performance could be inferred from the normalized CBF approach. While similarly predictive, the relationship between the SSM subject scaling factors and speech rate was more abstract and the relationship between speech rate and CBF was less apparent.

The initial observation in normal speakers (Sidtis et al., 2003) was replicated in a different group of normal speakers in a different scanner (Sidtis et al., 2018) using the normalized CBF approach. This pattern was also identified in three genotypes of hereditary spinocerebellar ataxia (Sidtis et al., 2006, 2010), in an impaired form in individuals with Parkinson’s disease not treated with deep brain stimulation (DBS), and in a modified form in individuals with Parkinson’s disease treated with DBS of the subthalamic nucleus (Sidtis et al., 2021).

The present study will contrast the lateralized linear regression model coefficients for the regions that predicted speech rate with the normalized CBF values for those regions and their opposite hemisphere homologs. The actual CBF values are mostly unpublished. The results suggest that functional meta-networks may orchestrate the extent to which the activity of specific regions, as measured by CBF, contribute to neurological systems responsible for specific behaviors.

## Materials and Methods

### Subjects

Subjects are described in the relevant primary publications. Subjects were right-handed, native speakers of American English. Brief summaries follow. The normal Minnesota speakers were age-matched to the ataxic Minnesota speakers. The ages of these two groups were not significantly different (independent *t*-test). The normal New York speakers were age matched to the PD and PD-DBS speakers. The ages of these three groups were not significantly different (independent *t*-test). Because of the relative demographics of the ataxic and PD speakers, the New York normal group was significantly older than the Minnesota normal group [*t* (27) = 3.59; *p* = 0.001].

#### Normal Speakers–Minnesota Study

Thirteen subjects (8 females, 5 males) had a mean age of 43 ± 11 years.

#### Normal Speakers–New York Study

Sixteen subjects (9 females, 7 males) had a mean age of 57 ± 10 years.

#### Ataxic Speakers–Minnesota Study

Twenty-four subjects (10 females, 14 males) had a mean age of 41 ± 17 years. There were 9 spinocerebellar ataxia type 1 subjects (SCA1), 8 spinocerebellar ataxia type 5 subjects (SCA5), and 5 spinocerebellar ataxia type 6 subjects (SCA6).

#### Parkinson’s Speakers–New York Study

Seven subjects with idiopathic PD (4 females, 3 males) had a mean age of 63 ± 10 years.

#### Deep Brain Stimulation Parkinson’s Speakers–New York Study

Seven subjects with idiopathic PD (males) who were treated with bilateral stimulation of the subthalamic nuclei, mean age of 57 ± 5 years, were studied twice. One study was conducted with the stimulators turned on at their usual clinical settings. The other study was conducted with the stimulators turned off.

### Positron Emission Tomography Imaging Procedures

#### Normal Speakers–Minnesota Study

Subjects were asked to repeat the syllable sequence/*pa-ta-ka*/as quickly as possible for 1 min during each of four H_2_^15^O PET scans.

#### Normal Speakers–New York Study

Subjects were asked to perform three different speech repetition tasks as quickly as possible in separate scans: the syllable/*pa*/, the syllable sequence/*pa-ta-ka*/, and the sentence/*pop the top cop*/. Each task was performed twice, each for 1 min during each of six H_2_^15^O PET scans.

#### Ataxic Speakers–Minnesota Study

As with the Minnesota normal speakers, subjects were asked to repeat the syllable sequence/*pa-ta-ka*/as quickly as possible for 1 min during each of four H_2_^15^O PET scans.

#### Parkinson’s Speakers–New York Study

Subjects with Parkinson’s disease were studied using the same protocol employed in the study of normal volunteers in the New York Study. Their Parkinson’s disease was treated pharmacologically without DBS. Subjects were asked to perform three different speech repetition tasks as quickly as possible in separate scans: the syllable/*pa*/, the syllable sequence/*pa-ta-ka*/, and the sentence/*pop the top cop*/. Each task was performed twice, each for 1 min during each of six H_2_^15^O PET scans.

#### Deep Brain Stimulation Parkinson’s Speakers–New York Study

Subjects with Parkinson’s disease treated with DBS were studied using the same protocol employed in the study of normal and Parkinson’s speakers in New York.

### Positron Emission Tomography Image Acquisition and Analysis

As with the subject descriptions, the methods are described in the relevant primary publications. Briefly, both the Minnesota and New York studies utilized bolus injections of H_2_^15^O to measure CBF. The Minnesota studies (Siemens-ECAT 953B) engaged the subjects in the speech task for 60 s beginning with the tracer injection allowing 10–15 s of involvement prior to the arrival of the tracer in the brain. The duration of each scan was 90 s. The New York studies (GE Advanced Tomograph) also engaged the subjects for 60 s. The delay time between tracer injection and brain activity was determined by a preliminary scan. Using the calculated delay time, tasks were initiated 15 s prior to detection of brain activity by the scanner. Image acquisition lasted approximately 2 min.

Scans were aligned within a subject and spatially normalized to standard space. A set of 22 (11 left, 11 right) regions of interest (ROIs) were applied to each scan. In the New York subjects, the 22 regions included sub-regions on adjacent levels. The ROIs were based on a preliminary study of speech and speech-like tasks (Sidtis et al., 1999). The sizes of the ROIs were larger than the areas of interest but did not extend beyond the relevant anatomical region. In the Minnesota data, ROIs were thresholded to voxels representing the upper 25% of signal to reduce partial volume errors and accommodate individual anatomic variability. In the New York data the threshold was the upper 10%. In all studies, CBF was normalized across subjects to a common global mean to reduce inter-subject variability in these relative rather than absolute regional data. Since these studies were not strictly quantitative (arterial blood isotope concentrations were not measured), normalizing each subject’s regional data using the ratio of each subject’s global mean CBF and the highest global CBF mean in the data set reduces inter-subject global differences, which are not readily interpretable.

### Statistical Analysis

Possible differences in left-right pairs of regions were examined using within-subject *t*-tests. The performance-based analysis utilized a stepwise multiple linear regression to determine if there was a combination of regional normalized CBF values that could predict speech rate (syllables per second) during scanning. The following criteria were used for all regression analyses: probability of *F* to enter (0.05), probability of *F* to remove (0.10), and tolerance (0.01). While over-fitting and under-fitting regression models can be a concern with this approach, cross-validation is recommended as a confirmatory procedure. The prediction of speech rate provided a cross-validation of the stepwise multiple regression analysis in each of the studies reported in this paper. Significantly, the brain regions identified using the stepwise multiple liner regression replicated the effects of brain lesions to these areas in clinical studies, supporting the validity of the analysis. Age was not used as a factor in the analyses. As noted, the Minnesota group and the New York groups were matched for age within their respective clinical groups (ataxia, PD, PD-DBS). The sample sizes for the clinical groups were determined by clinical criteria, availability, and resource. Consequently the groups were not large enough to consider sex as a factor.

## Results

### Normal Speakers–Minnesota Study

**FIGURE 1:**
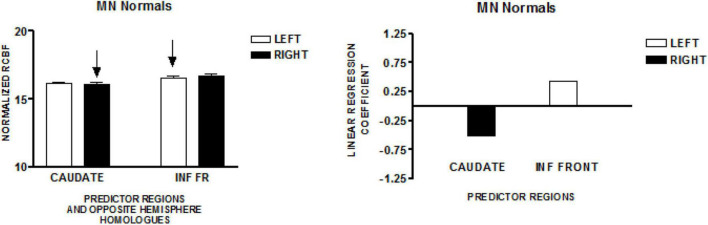
The results from a study of speech production in a group of normal volunteers in Minnesota (MN). The graph on the left presents the mean normalized CBF (± SEM) for the head of the caudate nuclei, and the inferior frontal region, bilaterally. Neither homologous pair has a significant left–right difference. The regions that predict speech rate are indicated by arrows. The graph on the right presents the linear regression weights for the two regions that predict speech rate (Sidtis et al., 2003).

The graph on the left in Figure 1 presents the mean normalized CBF for the head of the caudate nuclei, and the inferior frontal region, bilaterally. Neither homologous pair demonstrated significant left–right differences (paired *t*-test). The regions that predicted speech rate are indicated by arrows. The graph on the right of Figure 1 presents the linear regression weights for the two regions that predicted speech rate [*F*_(2,49)_ = 10.26; *p* = 0.0002]. The standardized coefficient value is −3.55 for the right caudate and + 2.51 for the left inferior frontal region (Sidtis et al., 2003).

### Normal Speakers–New York Study

**FIGURE 2:**
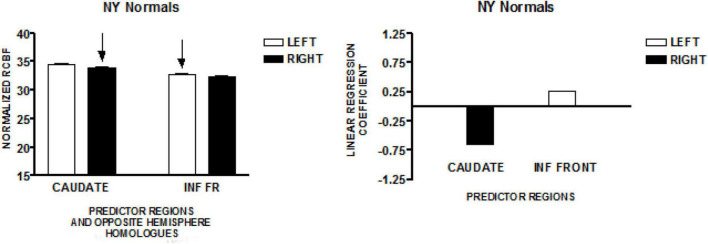
The results from a similar study of speech production in a group of normal volunteers in New York (NY). The graph on the left presents the mean normalized CBF (± SEM) for the head of the caudate nuclei, and the inferior frontal region, bilaterally. The graph on the right presents the linear regression weights for the two predictor regions (Sidtis et al., 2018).

The graph on the left of Figure 2 presents the mean normalized CBF values for the head of the caudate nuclei, and the inferior frontal region, bilaterally. Neither homologous pair demonstrated significant left-right differences (paired *t*-test). The regions that predicted speech rate are indicated by arrows. The graph on the right of Figure 2 presents the linear regression weights for the two regions that predicted speech rate [*F*_(2,89)_ = 5.09; *p* < 0.001]. The standardized coefficient value is −0.65 for the right caudate and + 0.27 for the left inferior frontal region (Sidtis et al., 2018).

### Ataxic Speakers–Minnesota Study

**FIGURE 3:**
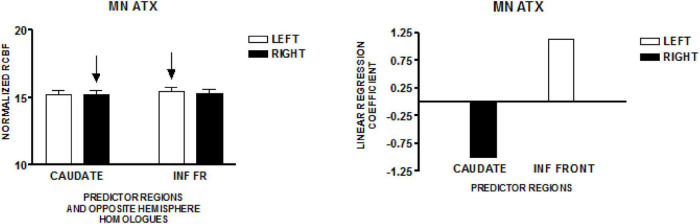
The results from a comparable speech production study in a group of individuals with hereditary spino-cerebellar ataxia using the same protocol employed in the study of normal volunteers in Minnesota (MN). The graph on the left presents the mean normalized rCBF (± SEM) for the head of the caudate nuclei, and the inferior frontal region, bilaterally. Neither homologous pair has a significant left–right difference. The regions that predict speech rate are indicated by arrows. The graph on the right presents the linear regression weights for the two regions that predict speech rate (Sidtis et al., 2006).

The graph on the left side of Figure 3 presents the mean normalized CBF for the head of the caudate nuclei, and the inferior frontal region, bilaterally. Neither homologous pair demonstrated significant left-right differences (paired *t*-test). The regions that predicted speech rate are indicated by arrows. The graph on the right presents the linear regression weights for the two regions that predicted speech rate [*F*_(4,119)_ = 21.82; *p* < 0.001]. The standardized coefficient value is −0.99 for the right caudate and + 1.13 for the left inferior frontal region (Sidtis et al., 2006).

### Parkinson’s Speakers–New York Study

**FIGURE 4:**
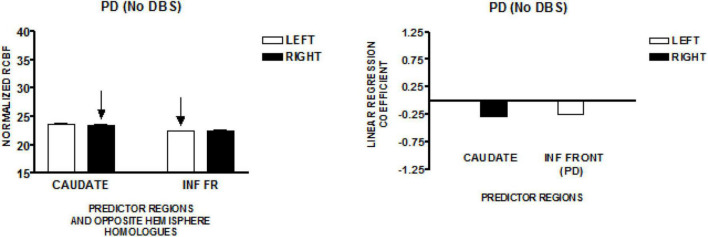
The results from a speech production study in individuals with Parkinson’s disease using the same protocol employed in the study of normal volunteers in New York (NY). Their Parkinson’s disease was treated pharmacologically without deep brain stimulation (DBS). The graph on the left presents the mean normalized CBF (± SEM) for the head of the caudate nuclei, and the inferior frontal region, bilaterally. The graph on the right presents the linear regression weights for the two predictor regions (Sidtis et al., 2021). The negatively weighted left inferior frontal region is adjacent and inferior to the left inferior predictor region in the NY normal group.

The graph on the left side of Figure 4 presents the mean normalized CBF for the head of the caudate nuclei, and the inferior frontal region, bilaterally. Neither homologous pair demonstrated significant left–right differences (paired *t*-test). The regions that predict speech rate are indicated by arrows. The graph on the right presents the linear regression weights for the two regions that predict speech rate [*F*_(2,81)_ = 6.87; *p* = 0.002]. The standardized coefficient value is −0.29 for the right caudate and–0.24 for the left inferior frontal region (Sidtis et al., 2021). The negatively weighted left inferior frontal region is adjacent and inferior to the positively weighted left inferior region found in the New York normal group. This difference may reflect an abnormal condition created as a consequence of Parkinson’s disease.

### Deep Brain Stimulation Parkinson’s Speakers–New York Study

**FIGURE 5:**
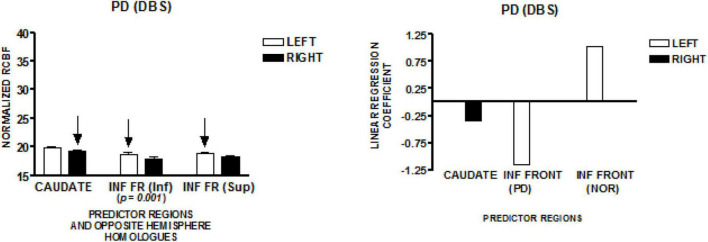
The results from a speech production study in individuals with Parkinson’s disease treated with deep brain stimulation (DBS) using the same protocol employed in the study of normal volunteers and Parkinson’s disease in New York (NY). The graph on the left presents the mean normalized CBF (± SEM) for the head of the caudate nuclei, and two inferior frontal regions bilaterally. The homologous left–right pair at the lowest anatomic level of the inferior frontal region demonstrated a left greater than right normalized CBF. The graph on the right presents the linear regression weights for the three regions that predict speech rate. The negatively weighted inferior frontal region is the same region that was negatively weighted in the Parkinson’s group without DBS. The inferior frontal region that was positively weighted is the same region that was positively weighted in the NY normal group. It is interesting to note that the significant left greater than right CBF in the lower inferior frontal region is negatively weighted in the regression model (Sidtis et al., 2021).

The graph on the left of Figure 5 presents the mean normalized CBF for the head of the caudate nuclei, and two inferior frontal regions, bilaterally. The homologous left-right pair at the lowest level of the inferior frontal region demonstrated a left greater than right normalized CBF [*t* (41) = 3.71; *p* = 0.001]. The regions that predict speech rate are indicated by arrows. The graph on the right of Figure 5 presents the linear regression weights for the three regions that predicted speech rate [*F*_(4,37)_ = 10.08; *p* < 0.001]. The standardized coefficient value is −0.34 for the right caudate, −1.15 for the lower left inferior frontal region, and + 1.04 for the more superior left inferior frontal region. The negatively weighted left inferior region is the same region found to be negatively weighted in the Parkinson’s disease group who were not treated with DBS. The positively weighted inferior frontal region is the same region found in the New York normal group. It is interesting to note that the significant left greater than right CBF in the lower inferior frontal region is negatively weighted in the regression model (Sidtis et al., 2021). This may reflect an effect of DBS.

## Discussion

The present study shows that a simple, multiple linear regression analysis reliably identified a reproducible, lateralized, cortical-subcortical CBF network that predicts speech rate during scanning. Importantly, the predictor regions are consistent with decades of clinical observations following the effects of focal brain lesions. In contrast to the performance-based regression model, normalized CBF values in the predictor regions and their opposite hemisphere homologs did not show significant left/right asymmetries (except for a single region in the presence of DBS). These examples argue that CBF alone does not correspond to actual performance.

Phenomenologically, the observations that CBF responses change with practice support the non-veridicality between CBF and behavior. Increases, decreases, and changes in the topography of CBF with practice may or may not reflect functional re-organization (Kelly and Garavan, 2005), but they do suggest other factors are in play in the relationship between CBF and behavior. Similarly, the observations that the patterns of CBF during a resting state mimic the patterns of the tasks with which the resting states are paired further suggests the influence of other factors in the relationships between CBF and performance (Sidtis et al., 2004; Hasson et al., 2009).

Simplified, the causal chain that is often assumed to link mental and behavioral activity to functional imaging signals is as follows: mental and behavioral planning and execution require increased neuronal activity, which requires increased metabolism. In turn, increased metabolism requires increased CBF. Realistically, however, the relationship between CBF and mental and physical activity is more like a tapestry than a chain.

Physiologically, the assumption of tight coupling between neuronal metabolism and CBF is generally true but often overstated. Fox and Raichle (1986) found that while CBF and oxidative metabolism were coupled during a resting state, uncoupling occurred during somatosensory stimulation. Even during the resting state, coupling between CBF and glucose metabolism is not uniform across brain regions. While some regions are coupled, others demonstrate hyper- or hypoperfusion (Gur et al., 2009).

The absence of a uniform relationship between CBF and brain function across regions is also demonstrated in the clinical phenomena of selectivity vulnerability to hypoxic-ischemic damage (Pulsinelli et al., 1982; Heiss, 1992; Schmidt-Kastner, 2015). Different regions of the brain can survive an ischemic event better than others. Selective vulnerability to ischemia as well as regional variability in the relationships between CBF and metabolism demonstrate that from a measurement perspective, the scale properties of CBF with respect to brain functions vary significantly across the brain as well as across scanning conditions, potentially causing difficulties for some types of analyses.

The tapestry analogy characterizing the relationship between CBF and brain activity best represents the role of the neurovascular unit (Drake and Iadecola, 2007). The neurovascular unit consists of multiple factors that work to control CBF: neurotransmitters and other vasoactive substances that can induce vasodilation and vasoconstriction, and the influence of non-neuronal cell lines. Astrocytes, with their connections to both synapses and cerebral blood vessels, play a significant role in the control of CBF (Iadecola and Nedergaard, 2007). The identification of diversity in the many factors that contribute to neurovascular control across the cerebrovascular network further suggests that rather than being referred to as a unit, the term neurovascular complex is more appropriate (Schaeffer and Iadecola, 2021).

It can be strongly argued that analyzing functional brain images based on CBF (or its magnetic resonance imaging surrogates) alone without a significant contribution of a relevant performance measure faces multiple hurdles in establishing specific brain-behavior relationships. Among the hurdles are the previously mentioned heterogeneous relationships between CBF and metabolism, differences in the relationships between CBF and brain tissue in different regions, and the complex, multi-factorial role of the neurovascular complex. Van Lancker Sidtis (2007) argued that uncertainty regarding the use of imaging signals as a dependent variable has been one of the factors that has limited the contribution of this technique to advancing neurolinguistics. The complexity of the relationship between CBF and brain activity requires more than simple stimulus-response thinking.

As subjects produced spoken speech in this study, the issue of respiration was raised as a possible factor. The role of volitional respiratory effort has been explored previously. Colebatch et al. (1991) found that targeted breathing resulted in increased CBF in the primary motor cortex, bilaterally, the right pre-motor cortex, the supplementary motor area and the cerebellum. Ramsay et al. (1993) similarly found that active inspiration and expiration were associated with bilateral CBF increases in the same regions. In a study of motor-to-sensory interactions, Paus et al. (1996) had subjects produce whispered syllables at different rates in the presence of bilateral white noise. They found a negative correlation between lip movements and respiration rates. After removing modeled subject and respiration effects, a number of regions were positively associated with lip movements including the left central sulcus as well as auditory and visual cortex. Associations between lip movements and CBF were not observed in any of the predictor regions identified in the present study.

Without question, respiration is part of speaking and is an integral part of the process. Previous efforts to isolate the phonation and articulation components of the production of the syllables used in this study using subtraction and addition did not produce neurologically meaningful results (Sidtis et al., 1999). In any case, the areas identified in these studies of respiration did not include the regions that predicted speech rate in the present study.

The present findings argue that the extent to which regional CBF is orchestrated to support behavior requires a higher level of organization than just a pattern of CBF. Functional meta-networks are suggested as a framework for super-ordinate organizational principles. Such meta-networks appear to operate by selectively amplifying or attenuating the contribution of selected regions in support of a specific function. The performance-based analyses used in these studies, as well as the image pre-processing, have been intentionally kept simple as the functional significance of CBF is neither uniform nor known across all brain regions and all scanning conditions. More complex analytic processes are not precluded but they should follow as better understanding of brain-behavior relationships emerge. Processing the connectivity of CBF activity alone does tell us something about the brain, but it does not necessarily provide insight into specific mental or behavioral operations in anything other than a general fashion. Friston (2011) characterized a dichotomy in the field of functional imaging that he described as functional segregation (i.e., localization) vs. integration (functional connectivity) suggesting that they were generally at odds. Such an opposing dichotomy is unnecessary as performance-based analyses have the potential for bridging this dichotomy by addressing both localization and connectivity for specific mental and behavioral operations.

In addition to potentially providing insights into specific mental and behavioral processes, functional meta-networks may better reflect some neuropathological conditions than primary networks. While not necessarily functional meta-networks, the differences in correlation patterns between speech predictor regions and non-predictor regions in the spinocerebellar ataxias occur as a function of genotype (Sidtis and Gomez, 2021). At the very least, these genotypic differences suggest the obvious: simple networks involved in specific behaviors operate in a broader neurological context. Reliable, clinically consistent networks for specific behaviors are a building block, not an endpoint. In this essay, a functional meta-network is inferred from multiple observations that weighted CBF from selected regions, rather than CBF alone, is significantly associated with a specific performance measure, and that the identified predictive regions are consistent with decades of clinical observations. The physiological mechanisms for functional meta-networks have yet to be defined, but it is likely that multiple layers of super-ordinate, functional meta-networks will have to identified to represent the brain’s activity during complex mentation and behaviors.

While the present studies replicate the involvement of two common regions and their valences across studies, the actual regression weights varied across study groups. In the future, larger studies would be helpful to establish confidence intervals for the predictors and more specific information about reproducibility. General metrics of regional involvement in specific tasks may represent the current limits of imaging ability. They should provide a sound foundation for describing specific functional networks in greater detail.

Regarding the pursuit of specific meta-networks, we have examined the correlations between speech rate predictor regions and the remaining regions in the data set. Perhaps a next stage would be to examine the linear regression models of the relationships between predictor and non-predictor regions. The introduction of the notion of meta-networks does not imply some non-neurophysiological mechanism. Rather, it suggests that the brain-behavior onion must be carefully peeled in layers. The problem for investigators is to determine the appropriate layers.

## Data Availability Statement

The raw data supporting the conclusions of this article will be made available by the authors, without undue reservation.

## Ethics Statement

The studies involving human participants were reviewed and approved by the Institutional Review Boards: University of Minnesota Medical School; Minneapolis Veterans Affairs Medical Center; Nathan Kline Institute for Psychiatric Research; Mount Sinai Medical Center; North Shore (Northwell) University Hospital. The patients/participants provided their written informed consent to participate in this study.

## Author Contributions

The author was solely responsible for the analyses and preparation of this manuscript.

## Conflict of Interest

The author declares that the research was conducted in the absence of any commercial or financial relationships that could be construed as a potential conflict of interest.

## Publisher’s Note

All claims expressed in this article are solely those of the authors and do not necessarily represent those of their affiliated organizations, or those of the publisher, the editors and the reviewers. Any product that may be evaluated in this article, or claim that may be made by its manufacturer, is not guaranteed or endorsed by the publisher.
